# Comparison of Prevalence of Psychiatric Disorders Among the Adult Population in Bangladesh, Before and During the COVID-19 Lockdown

**DOI:** 10.1192/bjo.2022.236

**Published:** 2022-06-20

**Authors:** Rifat Binte Radwan, Chiro Islam Mallik, Grace Pike

**Affiliations:** 1 The Crystal Centre, Essex Partnership University NHS Foundation Trust, Chelmsford, United Kingdom; 2 The Beacon Centre, Barnet, Enfield and Haringey Mental Health NHS Trust, Edgware, United Kingdom; 3 The Whiteleaf Centre, Oxford Health NHS Foundation Trust, Aylesbury, United Kingdom

## Abstract

**Aims:**

The aims were to determine and compare the prevalence of possible psychiatric disorders among Bangladeshi adults before and during lockdown. It was hypothesized that prevalence of possible psychiatric disorders would increase during the lockdown. In Bangladesh, lockdown was implemented in response to the COVID-19 pandemic resulting in conditions where those prone to developing psychiatric disorders were more vulnerable in an environment where the mental healthcare infrastructure is already lacking. Although many studies outlined the devastating impact on mental health that the lockdown measures created, this unique study specifically uses a World Health Organization developed research instrument for a lower-middle-income country.

**Methods:**

This was a cross-sectional, descriptive, comparative study with one stage design to determine possible psychiatric cases. Initially, 603 adults were randomly contacted using Facebook messenger & groups and email. Questionnaires including the validated Self Reporting Questionnaire (SRQ)-20 in Bangla, for screening psychopathology of the cases, and a structured questionnaire containing socio-demographic and other related variables, were inputted into Google Forms and hyperlinks were distributed. Eventually, 570 participants, from 18 to 77 years, with Internet access, who completed the questionnaires, were included in the study through purposive and consecutive sampling. The SRQ variables were divided into four categories: (1) depressive/anxious; (2) somatic symptoms; (3) reduced vital energy; and (4) depressive thoughts. Using IBM SPSS Statistics, paired sample t-tests were used during data analysis.

**Results:**

The mean age of cases was 34.69 ± 13.02 years; male: female = 1.41:1. The prevalence of possible psychiatric disorders was 43.9% during lockdown compared to 23.3% before lockdown (t = 19.497, P = 0.000). Before lockdown, sex and employment status were significant factors for the SRQ positive cases. After lockdown, in the SRQ positive cases, sex, educational status, COVID-19 positive cases and death due to COVID-19 among family members were highly significant (p = 0.0001) factors. Somatic symptoms and depressive thoughts were approximately double in prevalence among the SRQ positive cases during lockdown compared to before lockdown.

**Conclusion:**

There was a significant impact on mental health where a reduction in psychological and socioeconomic support occurred. These findings are in line with those in the literature where somatic symptoms have been identified as most commonly experienced during the pandemic. Increased depressive thoughts are associated with increased feelings of possible impending death and fear of an uncertain situation. Clearly, the mental health infrastructure of Bangladesh is in even greater need of rapid change to ensure resilience to the survivors of the lockdown.

